# Towards social curative psychedelic treatment

**DOI:** 10.1007/s44192-026-00426-3

**Published:** 2026-03-21

**Authors:** Martha Newson, Leor Roseman, S. Alexander Haslam

**Affiliations:** 1 Institute of Lifecourse Development, University of Greenwich, London, UK; 2 Centre for the Study of Social Cohesion, University of Oxford, Oxford, UK; 3 Department of Psychology, University of Exeter, Exeter, UK; 4 School of Psychology, University of Queensland, St Lucia, Australia

## Introduction

Recent advances in psychedelic-assisted therapies highlight their efficacy in treating various mental health conditions. Nevertheless, the fact that delivery is generally individualised may limit therapeutic outcomes by failing to capitalise on opportunities to build and harness a sense of shared social identity among participants in ways hypothesised by ‘social cure’ research. In line with this approach, evidence suggests that group-based interventions generally help to foster empathy, connectedness, and social functioning. Accordingly, integrating “social cure” principles informed by social identity theorising into treatment has the potential to enhance therapeutic efficacy by promoting interconnectedness within a supportive group setting.

Building on these insights, this article makes the case for integrating these two approaches to mental health treatment, which have hitherto been advanced in parallel within separate spheres of psychiatry. This speaks to the problem that while research into group-based therapies is burgeoning, it still lacks comprehensive theoretical underpinning [1]. This collaboration will also be crucial for the development of guidelines to ensure responsible and effective implementation of socially informed psychedelic therapies, in a way that ushers in a more comprehensive and socially embedded approach to mental health care.

Over the last decade there has been an upsurge of interest in exploring the capacity for psychedelic-assisted therapies to treat mental health conditions. In particular, these therapies have shown promise in the treatment of anxiety, depression, PTSD, and existential distress [2]. Indeed, in some instances their effects have been observed to surpass conventional psychopharmacological and psychotherapeutic interventions (see [3] for a robust meta-analysis). However, despite an emerging trend oriented towards group-based models, the majority of these therapies are currently delivered in individualised settings that diverge from the naturalistic group-based approaches that generally characterise delivery in indigenous, retreat-based, or underground contexts [4–8]. We advocate for the exploration of potential benefits of incorporating these social and group elements into psychedelic therapies and—building on long term research into psychedelic-assisted group therapy [9]—point to the social cure model as a framework for achieving this [1].

## Social cure as antidote to individualised treatment

As things stand, hesitancy to adopt group-based approaches is often a reflection of legal and professional concerns related to the desire to reduce the perceived risks of psychedelic treatments, where regulatory frameworks and clinical guidelines prioritise safety, standardisation, and clear lines of responsibility [3, 10]. This hesitancy is also reinforced by a biomedical paradigm which valorises randomised controlled trials and individual-level treatment effects as the gold standard of evidence, often encouraging the isolation of variables in order to demonstrate efficacy [11]. As a consequence of this, researchers and clinicians typically perceive social and contextual factors as ‘noise’ in ways that mitigate against an appreciation of their contribution to therapeutic outcomes. Yet a growing body of research investigating psychotherapy, group interventions, and social identity approaches to health indicates that relational processes, shared identity, and group belonging can be central mechanisms of therapeutic change rather than peripheral ‘noise’ [12–14]. Recognising these processes as integral to outcomes may therefore be essential for fully understanding and optimising the therapeutic potential of psychedelic-assisted interventions.

**Fig. 1:**
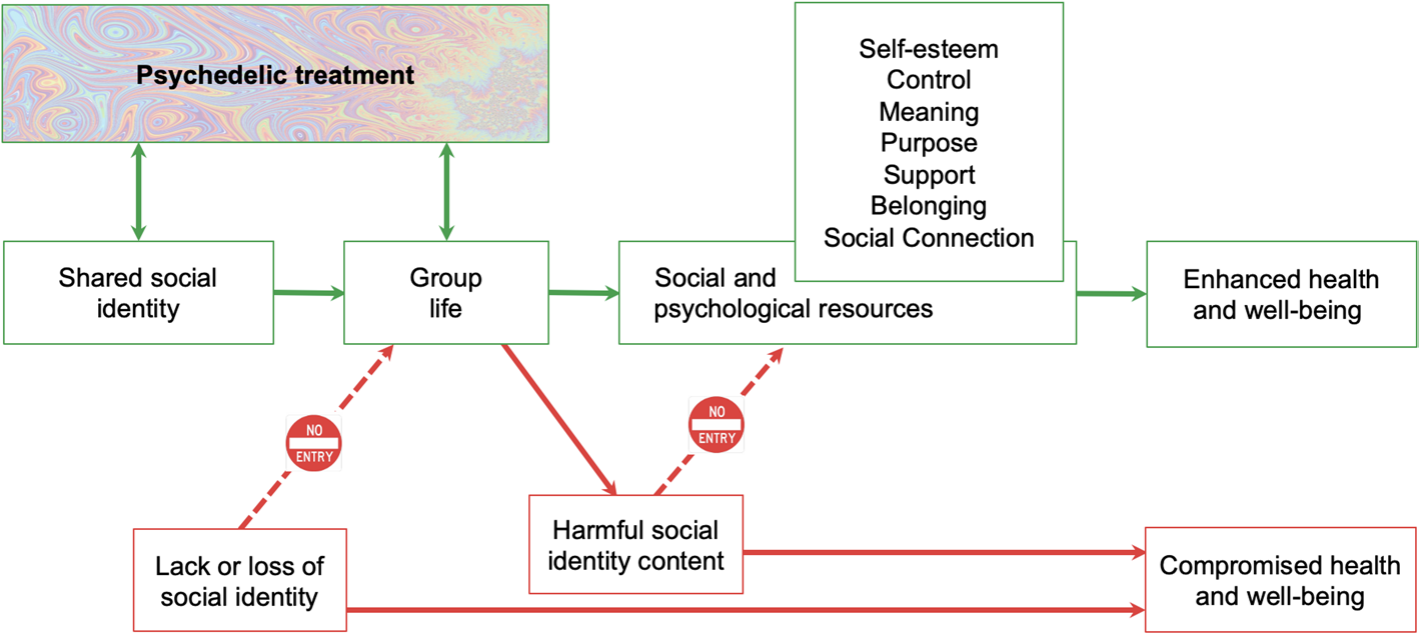
A model of the proposed integration of psychedelic and social cure approaches. Note: Green arrows denote social cure pathways, red arrows denote social curse pathway

As an antidote to these individualistic inclinations, we argue for the merits of an approach to psychedelic treatment that integrates this with a ‘social cure’ model that recognises the capacity for social identity processes and group dynamics to promote mental health [15]. This model is represented schematically in Fig. 1 and suggests that when individuals identify with a positive group (i.e., one whose norms, values and goals are the basis for positive social identity content) this gives them access to valuable social and psychological resources that support—and typically enhance—their overall well-being. These resources include social support and self-esteem as well as a sense of self-efficacy, control, purpose and meaning [12].

On top of this, social cure research has shown that the sense of belonging and group-based social connection that social identity creates can be therapeutic agents that promote mental health through collective experience [15]. This is seen, for example, in the positive sense of ‘we-ness’ that a person might gain from feeling themselves to be part of a football club, a work team, or a community-based support network. This perspective aligns with growing recognition of the importance of social connectedness and community for mental health and mental health promotion [13].

In this way, the subjective sense of collective self (or ‘we-ness’) that people derive from group membership, emerges as a crucial factor in the therapeutic process. It also follows that incorporating social identity principles into psychedelic therapy can encourage individuals to explore and embrace this expansive sense of self within the context of a supportive group. This can lead to increased self-awareness, personal growth, and a heightened sense of interconnectedness—all of which have been observed elsewhere to be fundamental aspects of the therapeutic benefits of group-based clinical interventions [15].

Equally, research indicates that psychedelics can enhance feelings of empathy, group bonding, connectedness, social cognition, and overall social functioning [10], in ways that synergetically support the work of the social cure. Consistent with these claims, underground practices, such as ‘raving’ suggest that group bonds and emotional sharing contribute to long-term changes in social connection [16], an effect also documented in longitudinal studies of guided retreats and ceremonies contributing to enhanced well-being [17]. Exploratory studies that incorporate group therapy sessions into indigenous psychedelic treatment have also been found to promote social cohesion and a sense of belonging that has beneficial consequences, especially when addressing trauma [18].

At the same time, though, it is apparent that the latter outcomes are dependent on features of the therapeutic set and setting and require there to be trust and rapport within the group therapeutic environment. Giving therapists the skills and experience to run group sessions is therefore extremely important, but equally it is important that they are attuned to principles of *identity leadership* through which they can create, advance, represent and embed a sense of shared social identity in the groups they work with [14]. When they do, there is evidence that this can be an effective way not only to build therapeutic alliance but also to leverage the benefits of group identities still maintaining safety and control [19–20].

## Pragmatics: ethics and financial implications

Before psychedelic group therapy can be integrated into mainstream psychiatric practice it is also going to be important to address ethical and legal dimentions. Ethically, practitioners must navigate the potential adverse effects of group dynamics. These relate to the ‘social curse’ that can flow from harmful social identity content (Fig. 1) and from culturally inappropriate adaptations of treatment. The ‘social curse’ refers to group processes undermining wellbeing when shared identities become exclusionary, reinforce harmful norms, or intensify social pressure [21]. These risks may be heightened in psychedelic contexts, where heightened suggestibility, emotional openness, and boundary permeability can amplify group influence, increasing vulnerability to conformity pressures, boundary violations, or the internalisation of maladaptive beliefs if facilitation and safeguards are inadequate [22]. Accordingly, identity leadership needs to pay careful attention to group composition, facilitator training, ethical safeguards, and culturally sensitive practice to ensure that collective processes support, rather than compromise, therapeutic outcomes [14, 23].

While emerging scholarship and community narratives often highlight the therapeutic and prosocial potential of collective altered-state experiences, the social context in which such states occur can also introduce risks. Group psychedelic practices may, under certain conditions, intensify conformity pressures, reinforce hierarchical authority structures, or foster identity fusion with charismatic leaders or fringe narratives [24–25]. Historical and ethnographic analyses also show that expectations, cultural framing, and social interaction can shape subjective experiences in ways that amplify shared belief systems and group norms [26]. These dynamics underscore the importance of recognising that collective altered states are not inherently beneficial and may, in some contexts, contribute to coercive influence or maladaptive group cohesion.

Early debates surrounding the long-term effects and methodological limitations of the Good Friday experiment (whereby theology students were administered psilocybin or an active placebo during a Good Friday service to assess whether psychedelics could occasion mystical experiences in a religious setting) have already highlighted the need to interpret group psychedelic experiences within their social and institutional contexts rather than as purely individual phenomena [27]. Accordingly, a fuller account of collective psychedelic experiences should acknowledge both beneficial and harmful outcomes and clarify how social identity processes and structured training protocols can be leveraged to prevent coercive dynamics, rather than merely explain them.

A possible pragmatic advantage to group-based psychedelic-assisted therapy may be cost efficiency and scalability, considering most current 1:1 or even 1:2 delivery models create significant resource constraints. Such benefits will need careful investigation, as cost advantages are not automatic: savings may be offset where additional facilitators, intensive screening, enhanced risk management, or specialised training are required to maintain safety in altered-state contexts. Indeed, we see the economic case for a social identity approach to rest more on the fact that it is an efficient and effective pathway to cure than that it saves time and effort.

The social cure model’s past successes make it well-equipped to help psychedelic practitioners extend into the complex terrain of group dynamics [15]. Similarly, legal and funding frameworks must evolve to accommodate the unique dynamics of group therapy, recognising its potential benefits while establishing safeguards to mitigate potential risks. Collaborative efforts between policymakers, mental health professionals, and researchers will therefore be crucial in shaping responsible guidelines for the ethical and legal administration of group-based psychedelic therapies. To what extent can the successes of the social cure literature, including global studies [12] and randomised control trials [28], expedite the integration of social dynamics into medical and scientific psychedelic practice?

## Conclusion

As psychedelic-assisted therapies continue to gain recognition and traction in mental health treatment, the need for innovative delivery models that harness the power of group dynamics is becoming increasingly apparent. Group-based psychedelic therapies, guided by the principles of social cure research, have the potential to enhance mental health care by addressing not only individual healing but also collective well-being [1]. More broadly, the predominance of individual delivery models may reflect wider structural and cultural orientations within hegemonic mental health care frameworks, where one-to-one therapy is normative despite the efficacy and potential cost-effectiveness of group approaches. Within these frameworks, psychedelic therapy may thus only amplify existing individualistic treatment norms, rather than originate them, pointing to the importance of cross-cultural research to determine when and for whom collective formats enhance safety, acceptability, and therapeutic outcomes.

If we are to pave the way to a more comprehensive and socially embedded approach to psychedelic-assisted mental health care, this paradigm shift will require collaborative efforts from researchers, clinicians, policymakers, and the broader mental health community to navigate challenging legal, ethical, and professional terrain. This, though, is no longer beyond the realms of our collective imagination. With sufficient collective will, there is exciting new territory to discover, map, and open up for those in need of access.

## Data Availability

No datasets were generated or analysed during the current study.
